# Applications of a vacuum-actuated multi-material hybrid soft gripper: lessons learnt from RoboSoft manipulation challenge

**DOI:** 10.3389/frobt.2024.1356692

**Published:** 2024-05-28

**Authors:** Saikrishna Dontu, Elgar Kanhere, Thileepan Stalin, Audelia Gumarus Dharmawan, Chidanand Hegde, Jiangtao Su, Xiaodong Chen, Shlomo Magdassi, Gim Song Soh, Pablo Valdivia Y. Alvarado

**Affiliations:** ^1^ Digital Manufacturing and Design Centre, Singapore University of Technology and Design, Singapore, Singapore; ^2^ Singapore-HUJ Alliance for Research and Enterprise (SHARE), The Smart Grippers for Soft Robotics (SGSR) Programme, Campus for Research Excellence and Technological Enterprise (CREATE), Singapore, Singapore; ^3^ Engineering Product Development Pillar, Singapore University of Technology and Design, Singapore, Singapore; ^4^ Robotics Innovation Laboratory, Singapore University of Technology and Design, Singapore, Singapore

**Keywords:** soft gripper, vacuum actuation, RoboSoft competition, manipulation, food handling, lab to market, bio inspiration

## Abstract

Soft grippers are garnering increasing attention for their adeptness in conforming to diverse objects, particularly delicate items, without warranting precise force control. This attribute proves especially beneficial in unstructured environments and dynamic tasks such as food handling. Human hands, owing to their elevated dexterity and precise motor control, exhibit the ability to delicately manipulate complex food items, such as small or fragile objects, by dynamically adjusting their grasping configurations. Furthermore, with their rich sensory receptors and hand-eye coordination that provide valuable information involving the texture and form factor, real-time adjustments to avoid damage or spill during food handling appear seamless. Despite numerous endeavors to replicate these capabilities through robotic solutions involving soft grippers, matching human performance remains a formidable engineering challenge. Robotic competitions serve as an invaluable platform for pushing the boundaries of manipulation capabilities, simultaneously offering insights into the adoption of these solutions across diverse domains, including food handling. Serving as a proxy for the future transition of robotic solutions from the laboratory to the market, these competitions simulate real-world challenges. Since 2021, our research group has actively participated in RoboSoft competitions, securing victories in the Manipulation track in 2022 and 2023. Our success was propelled by the utilization of a modified iteration of our Retractable Nails Soft Gripper (RNSG), tailored to meet the specific requirements of each task. The integration of sensors and collaborative manipulators further enhanced the gripper’s performance, facilitating the seamless execution of complex grasping tasks associated with food handling. This article encapsulates the experiential insights gained during the application of our highly versatile soft gripper in these competition environments.

## 1 Introduction

Robotics has played a pivotal role in addressing the challenge of a severe labor shortage, particularly through the automation of repetitive and unskilled tasks (Jungmittag and Pesole, 2019). In industries characterized by limited object geometries and well-defined specifications, tailored automation solutions have demonstrated the potential to significantly enhance efficiency and productivity. However, the applicability of this approach becomes less straightforward in dynamic sectors like food and agriculture (Caldwell et al., 2009). Here, the handling of delicate or squishy items poses significant challenges, compounded by the variability in parameters such as shape, size, stiffness, and orientation (Wang et al., 2022a). Customized solutions in such scenarios have a risk of failure incurring substantial losses when parameters change, and the complexity of preprogramming all conceivable possibilities, such as precise force control for delicate fruit handling, becomes a formidable task.

The escalating popularity of soft grippers stems from their inherent underactuation, obviating the need for meticulous force control and enabling adaptation to different object shapes and sizes due to their hyper elastic material properties (Shintake et al., 2018). Fabricated from soft materials, these grippers prove particularly suited for tasks involving delicate items, ensuring gentleness in handling—crucial in the context of food manipulation (Elfferich et al., 2022). Soft grippers are often categorized based on their mode of actuation such as pneumatic, cable driven, vacuum, shape memory alloy, electroactive polymer, electro adhesion, etc. “Pneunets” actuators which are one of the types of the pneumatic fingers because of their reliability and ease of fabrication have gained popularity and are widely employed (Zaidi et al., 2021). Notably, companies like ‘Soft Robotics Inc.’ have already deployed variants of these actuators in industries such as bakery, meat, and other fast-moving consumer goods (Miron et al., 2018). “Pneunets” actuators feature a series of inflatable chambers cast from soft elastomers, constraining the flat part of the actuator with an inextensible layer or a stiffer elastomer (Gariya and Kumar, 2022). Simple chamber inflation leads to actuator bending, and various gripper designs use these actuators in different configurations and numbers. However, the quest for higher gripping forces necessitates the use of elevated pressure ranges, posing safety concerns and increasing the risk of system failure (Jiao et al., 2019). Jamming grippers represent an innovative type of soft robotic actuators designed to effectively handle objects of arbitrary shapes (Fitzgerald et al., 2021). These devices operate by encasing granular material in a pliable outer membrane that is linked to a vacuum pump. To grasp an object, the gripper is first pressed against it in an unjammed state; then, the air is sucked out, causing the granules to lock together into a solid-like state and secure the object firmly. Despite their versatility, there’s still a significant amount of uncertainty surrounding the best shapes for the granular constituents, especially in relation to how these shapes interact with the forms of the objects being gripped, affecting the overall efficiency of the device in specific applications. Granular jamming, while advantageous in terms of fabrication simplicity and the ability to conform to a wide range of object geometries, introduces variability in gripping force dependent on the object’s contact surface (Gao et al., 2020; Wang et al., 2022b). Cable-driven mechanisms offer high forces but require high-torque motors to bend gripper fingers and risk system bulkiness (Artemiadis and Thalman, 2020).

Despite the progress in these technologies, their deployment into industries requires several iterations to enhance the technology readiness level before commercialization. One crucial requirement is pilot testing in real environments but doing so for individual technologies that are still in the research phase would be extremely challenging. Scenario-based robotic competitions emerge as a potent avenue to bridge this gap, offering a platform to demonstrate the capabilities of new technology as proof of concept (Downs et al., 2021). In this context, robotic competitions not only provide a benchmark for evaluating technology performance in solving challenging tasks but also contribute to the advancement and adoption of robotic solutions in industries such as food through events like IEEE ICRA^1^ and IEEE RoboSoft^2^. Additionally, competitions shed light on key aspects of practical applications, aiding the understanding of overall settings when deploying these technologies. The manipulation challenges held during the IEEE RoboSoft conferences aim to showcase novel soft robotics technologies applicable to various real-life grasping and manipulation scenarios. Our research group has previously worked on a vacuum-actuated hybrid soft gripper consisting of soft fingers mounted onto a soft palm which was able to lift parts as heavy as 2 kg (Subramaniam et al., 2020). Use of vacuum made the system safer compared to positive pressure devices, and also faster compared to cable driven approach as time is an important factor in competitions (Pustavrh et al., 2023). Our group used a modified version of our vacuum-actuated hybrid multi-material soft gripper (Jain et al., 2020a), tailored to the specified tasks of each year’s competition, maintaining a base design while incorporating additional independent actuators or sensors for enhanced functionality. The main contribution is the usage of the upgraded iterations of a base technology to suit different scenarios in practical competition environments which is a crucial part of the lab-to-market transition including the fabrication novelty of integrating the sensors to increase the functionality of the grippers. One such feature was the addition of a suction cup with cleats at the center of the palm to increase the payload capacity for handling heavy fruits such as watermelon and papaya in RoboSoft 2021. For RoboSoft 2023, hook-shaped nails were included to handle spaghetti noodles.

The paper is structured into main sections summarising design, methods, results, and learnings, with subsections dedicated to the respective RoboSoft competitions of 2021, 2022, and 2023. A table is provided for a high-level comparison of the grippers used in the respective RoboSoft competitions to the other state-of-the-art multipurpose grippers (Table 1) (Amend and Lipson, 2017; Choi et al., 2017; Chin et al., 2020; Wang et al., 2021; Alici and Richards, 2023; Zhu et al., 2023). The design section describes the elements of the vacuum-actuated hybrid soft gripper, emphasizing the specific features integrated to align with the competition tasks. In the methods section, the fabrication of gripper components and their assembly is detailed. In the case of RoboSoft 2023, an attempt to integrate the gripper with novel force and slip sensors was made along with using a depth camera for object localization. Furthermore, we discuss the experiments conducted before the competitions to test the system and the final competition results, followed by a conclusion outlining key learnings and future prospects.

**TABLE 1:** Comparison table for state-of-the-art multipurpose grippers Legend: Fully possible ✓✓ Partially possible ✓ Not possible ×.

Gripper	Payload (kg)	Actuators	Thin flat items	Multiple geometries	Granular items	Squishy items
RoboSoft 2021 Gripper	6	Soft Fingers, Active Palm, Fixed Nails, Suction Cup	✓✓	✓✓	✓	✓✓
RoboSoft 2022 Gripper	5	Soft Fingers, Fixed Palm, Fixed Nails, Suction Cup	✓✓	✓✓	✓	✓✓
RoboSoft 2023 Gripper	1.4	Soft Fingers, Active Palm, Fixed Nails	✓✓	✓✓	✓	✓✓
Gripper with Locking Mechanism (Alici and Richards, 2023)	10.2	Rigid Fingers with Flexible links, Locking Mechanism	×	✓	×	✓
JamHand (Amend and Lipson, 2017)	2.5	Two-fingered configuration with pockets of granular material in the fingertips	✓	✓✓	×	✓
Multipurpose Universal Gripper (Choi et al., 2017)	3	3-DOF fingers consist of the identical joint modules which include a driving motor, reduction gears	×	✓✓	×	✓
Gripper that integrates three modes of grasping: suction, parallel jaw, and soft fingers (Chin et al., 2020)	2.44	Handed shearing auxetic (HSA) fingers with suction cups at the tip	✓✓	✓✓	×	✓✓
Circular Shell Gripper (Wang et al., 2021)	5.2	A single module including a rigid shell and a soft chamber	×	✓✓	×	✓✓
Bioinspired Multimodal Multipose Hybrid Gripper (Zhu et al., 2023)	27	Gripper with fingers coupled in parallel by a rigid actuator based on an underactuated skeleton and a fiber-reinforced soft actuator	✓✓	✓✓	×	✓✓

## 2 Design criteria

**FIGURE 1:**
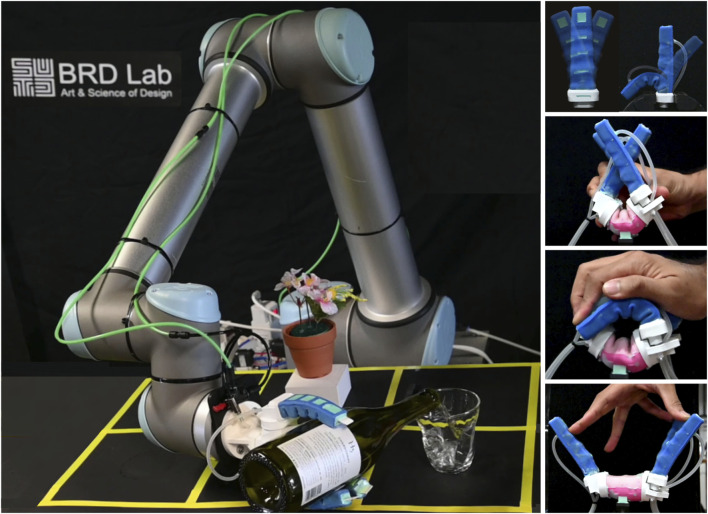
Emulation of a wine-pouring scenario using a robotic arm and a soft gripper.

All RoboSoft competitions mandated the demonstration of robot “softness”, which could be evaluated based on material softness or structural compliance. Softness was required to enhance the robot’s functionality and capabilities. Many tasks, such as handling food, pouring wine, or picking up delicate cookies, were designed to emulate real-world situations (Figure 1). For these tasks, an anthropomorphic gripper design was chosen, specifically a three-finger gripper that proved suitable for diverse shapes and grasping modes, as determined in our prior study (Subramaniam et al., 2020). Given that some competition details were undisclosed until the final day, extreme versatility was essential to accommodate last-minute changes, such as item location or type based on size or softness.

The vacuum-actuated hybrid soft gripper comprises two main components: soft fingers and a palm (Subramaniam et al., 2020). The soft fingers employ a multi-material design with stiff wedges encapsulated by a softer, thin skin. Vacuum actuation induces finger bending as the thin skin collapses between the wedges. The soft gripper palm shares a similar multi-material design, using vacuum actuation to collapse thin skin between stiffer wedges. This active palm enhances the gripper’s payload capacity while offering precise aperture control. The gripper prototype evolved from this baseline design to meet specifications and address the challenges presented in each competition. The design table summarizes the design features added to the gripper depending on tasks that could be handled (Table 2).

**TABLE 2:** Design Table for overall design components in RoboSoft competitions.

Competition	Objects to be handled	Object type	Grasping strategy	Design actuators
RoboSoft 2021	Watermelon	Large, heavy	Heavy payload exceeds the aperture limit of the active palm hence a suction cup with a holding force of 2 kg would aid in the lifting while fingers would reduce the vibrations during trajectory	Suction cup + Fingers
	Raspberry	Small, soft	The small size requires active palm for optimum gripper pose before picking along with nails for precision grasping	Active Palm + Nails
	25 mm coin	Small, thin, flat	The flatness requires active palm for optimum gripper pose before picking along with nails for precision grasping	Active Palm + Nails
RoboSoft 2022	Coke can	Medium, cylinder	Suction cup is ideal for smooth surface and fingers provide vibration reduction	Suction cup + Fingers
	Marshmallow	Small, soft	Soft fingers conform to the soft surface and the nails provide support	Fingers + Nails
	Grape	Small, delicate	Soft fingers conform to the soft surface and the nails provide support	Fingers + Nails
	Plant1	Medium, frustum	Tapered pot shape ensures that the fingers provide power grasp	Fingers
	Plant2	Medium, frustum	Tapered pot shape ensures that the fingers provide power grasp	Fingers
	Wine bottle	Medium, cylinder	Suction cup is ideal for bottle surface and fingers provide power grasp while reducing vibrations	Suction cup + Fingers
	Glass	Medium, frustum	Fingers conform to the glass rim while picking it up with power grasp	Fingers
RoboSoft 2023	Sausages	Long, cylinder	Soft fingers conform to the soft surface of the sausage	Fingers
	Broccoli	Small, delicate	Soft fingers conform to the soft surface of broccoli	Fingers
	Carrots	Small, thin, flat	Fingers and the palm provide a partial scooping grasp for slices along with the nails	Fingers + Nails + Active Palm
	Green Beans	Small, long, thin	Fingers and the palm provide a partial scooping grasp for stems along with the nails	Fingers + Nails + Active Palm
	Spaghetti Noodles	Delicate strands	Hook nail allows the scooping of noodles. Even a few strands would provide partial points	Fingers + Nails
	Cookies	Small, thin, flat	Nails of which one is hooked allows the grasping of the cookie boundary	Nails
	Fried eggs	Medium, soft, flat	Soft fingers along with nails provide a pinch grasp to pick the flat eggs	Fingers + Nails
	Ice gems	Small, granular	Fingers provide a scooping grasp for gems along with the nails	Fingers + Nails
	Orange juice	Medium, cylinder	Fingers provide power grasp for the bottle surface while reducing vibrations	Fingers

### 2.1 RoboSoft 2021

**FIGURE 2:**
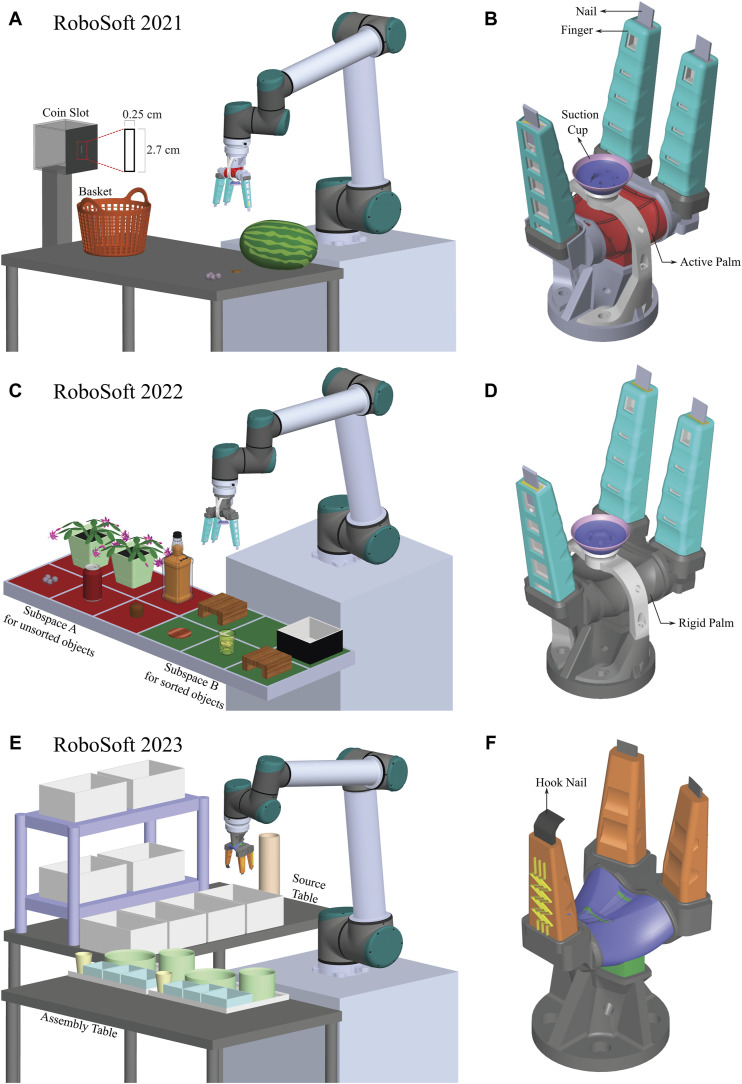
RoboSoft Layouts: **(A)** Layout of RoboSoft 2021 with four tasks of lifting a heavy, smooth-skinned fruit–watermelon, handling a small, delicate fruit–berry, picking up a coin on a hard surface, inserting the coin into the coin slot; **(B)** Gripper used in RoboSoft 2021 consisting of active palm for aperture control, suction cup for increased payload capacity and nails at fingertips for flat items; **(C)** Layout of RoboSoft 2022 for sorting items in Subspace A to Subspace B with three tasks of picking of three objects (grape, coke can, marshmallow) from Subspace A and placing them in a collecting basket in Subspace B, picking of two plant pots from Subspace A and placing them on two shelves in Subspace B; picking a wine bottle from Subspace A and pouring a dram of liquid into the glass in Subspace B along with picking up the glass and placing it on a coaster after safely placing the bottle back on the table; **(D)** Gripper used in RoboSoft 2022 consisting of 3D printed rigid palm with fixed aperture to avoid vibrations and spillage while handling wine and a suction cup for stable grasps; **(E)** Layout of RoboSoft 2023 with the task of assembling two food trays by picking the food items on shelves and bottle of orange juice from a source table to specific containers on the trays kept on an assembly table; **(F)** Gripper used in RoboSoft 2023 consisting of smaller fingers and active palm for fitting into limited shelf space, sensors integrated at finger tips for force control and slip detection, and a hook shaped nail for scooping noodles.

A three-round bracket format was used for the competition in 2021. Teams were eliminated in each phase, with the winners moving on to the semifinal and final rounds. Evaluation criteria included proving gripper abilities such as: (I) strength by lifting a heavy, smooth-skinned fruit weighing between 1 and 2 kg; (II) compliance by handling a small, delicate fruit; (III) precision by picking up a coin from a hard surface; and (IV) dexterity by dropping the coin into an appropriate slot (Figure 2A). Moreover, pick-and-place actions had to be separated by a minimum of 50 cm. Each job had a set time limit of 3 minutes, and the teams’ ability to finish quickly would determine the winner. Since autonomy criteria was not specified, some teams used teleoperation, while our team used waypoint programming. In the first task, our team concentrated on payload capacity and grasp stability, which were essential for moving large fruits, like watermelons, over a distance of 50 cm without dropping them due to high vibrations that could arise from acceleration. Our team used a suction cup to solve this challenge (Schmalz, 2022) which increased grasping stability and worked along the active palm and soft fingers to increase payload capacity (Tawk et al., 2019). The diameter of the suction cup of 3.5 cm was selected as per the payload capacity of 2 kg along with a safety factor of two using the below formula.

$$d=1.12\times \surd \frac{\left(m\times S\right)}{\left(P\times n\times \mu \right)}$$

where m (payload) is 2 kg; S (safety factor) is 2; P (vacuum) is 0.8 bar; n (number of suction cups) is 1; μ (coefficient of friction) is 0.5.

This calculation was one for the case of vertical pick-up to account for the vibrations occurring due to robotic arm acceleration which could lead to shear. Also, circular cleats were introduced on the internal surface of the suction cup to allow for more surface contact owing to their non-slip properties (Figures 2B, D).

A flat coin and small fragile fruits had to be handled in the remaining tasks. Our team used a novel actuator design with nails at the fingertips (Jain et al., 2023) and precise palm aperture control, drawing inspiration from the incredibly dexterous human hand (Figure 2B). To protect fragile fruits like grapes from getting bruised, soft padding was added to the nails. The thin coin, which had very little surface area available for grasping, presented a special level of difficulty, and the soft nail padding was essential to provide enough friction to facilitate coin grasping.

### 2.2 RoboSoft 2022

The 2022 competition introduced autonomy requirements, with penalties for non-compliance. Teams were judged based on overall points and successful scenario completion within 15 min. The scenario comprised Subspace A (red), housing unsorted initial items, and Subspace B (green), containing goal areas for the tasks, each divided into six smaller sections (Figure 2C). The target tasks included: (I) Picking three different items (grape, coke can, marshmallow) from Subspace A and placing them in a collecting basket in Subspace B, (II) Picking two plant pots from Subspace A and placing them on two small shelves in Subspace B, and (III) Picking a wine bottle from Subspace A, pouring a dram of liquid into a glass in Subspace B, and safely placing the bottle back on the table, followed by picking up the glass and placing it on a coaster. The gripper prototype used in RoboSoft 2021 proved suitable for the 2022 challenge. Independent additional actuators–active palm, suction cup, and nails, along with soft fingers, expanded the gripper’s workspace, making it multimodal and suitable for a highly dynamic environment. Using the suction cup for the smooth top metallic surface of the coke can, was effective while soft fingers successfully grasped delicate grape and spongy marshmallow. The active palm ended up playing a minor role as the aperture control for small items could be achieved by partial finger actuation. The suction cup and finger grip force proved sufficient for tasks involving plant pots and the wine bottle, thus rendering the active palm redundant. Moreover, the usage of the active soft palm during liquid pouring induced vibrations, risking spillage, and points deduction. Consequently, the active palm was replaced with a 3D-printed rigid palm with a fixed aperture to mitigate the risk of spillage and enhance the overall performance (Figure 2D).

### 2.3 RoboSoft 2023

The 2023 competition emphasized grasping strategies, system integration, autonomy, execution speed, and repeatability. The main task involved assembling up to two food trays within a 30-min timeframe. The scenario included a source table with food items on shelves and an assembly table holding trays with specific containers, featuring classic pick-and-place food assembly (Figure 2E). A list of 10 items, including eight known, one unknown, and orange juice, was provided before the competition. On the competition day, seven items were randomly selected to be assembled in destination containers along with pouring orange juice from a carafe into two cups on the assembly table before returning to the source table. The items included a diverse range of flat, delicate, and granular items, each requiring a suitable grasping strategy. Unlike previous competitions, there was no high payload capacity requirement as most items were small. However, the gripper needed to fit within the dimensions of the containers on the source table due to limited shelf space. Consequently, the suction cup was removed, and the gripper volume was significantly reduced from its previous version. Reduction in finger length minimized vibrations, crucial for grasping food items and avoiding slipping while moving at high speeds between tables. Other modifications included changing the nail shape from flat to curved (hook-like) for improved noodle scooping (Figure 2F). An optic force sensor at the fingertip counted successful grasps and controlled grasping force. Additionally, a strain gauge based tactile sensor was integrated onto another fingertip to measure slip conditions for different food items, determining the maximum robotic arm speed before slip onset. Finally, we also aimed to demonstrate autonomous behavior using computer vision and overall system integration for assembly tasks.

## 3 Materials and methods

### 3.1 Finger fabrication

**FIGURE 3:**
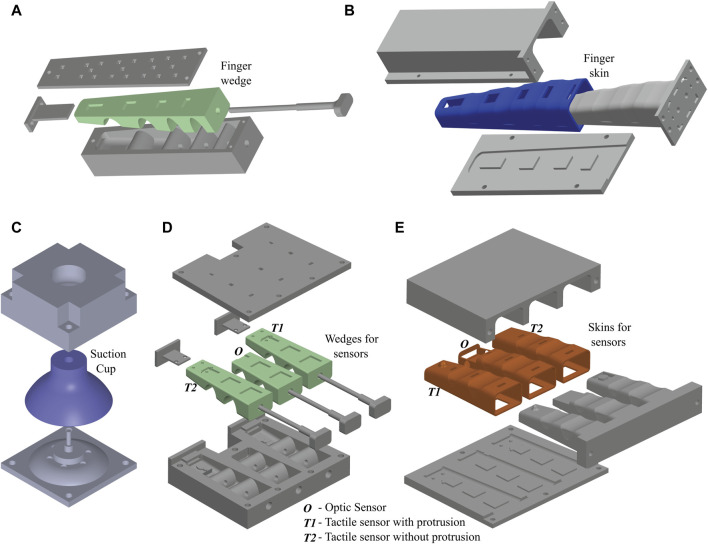
Fabrication of Gripper components: **(A)** Five stiff wedges cast using a four-piece 3D printed ASA (Acrylic Styrene Acrylonitrile) mold setup by dispensing Smooth-Sil 960 in the mold through compressed air-assisted injection molding; **(B)** The finger’s skin was cast by applying MoldStar 30 to a three-piece press mold to create a 0.5 mm layer which was later reinforced with a thin strain-limiting PTFE-coated fiberglass fabric sheet before casting another 0.5 mm layer; **(C)** Suction cup was cast using a two-piece mold setup by dispensing Smooth-Sil 960 in the mold through compressed air-assisted injection molding; **(D, E)** O denotes the wedge and skin cast for the finger holding optic sensor, T1 denotes the wedge and skin cast for the finger holding tactile sensor with protrusion at fingertip, and T2 denotes the wedge and skin cast for the finger holding tactile sensor entirely submerged in the wedge without any protrusion at fingertip.

Our soft grippers comprised three fingers and active palms, both designed with a multi-material hybrid approach. Each finger consisted of multiple stiff wedges cast using a four-piece 3D printed ASA (Acrylic Styrene Acrylonitrile) mold setup (Figure 3A). Smooth-Sil 960 (Smooth-On) was employed for mold filling through compressed air-assisted injection molding. ASA nails, 3D printed, were snugly inserted into the top wedge of each finger. For the finger’s skin, MoldStar 30 (Smooth-On) was manually applied to a three-piece press mold to create a 0.5 mm layer (Figure 3B). The finger’s skin was reinforced with a thin strain-limiting PTFE-coated fiberglass fabric sheet, embedded within to provide inextensibility while maintaining flexibility for bending. Subsequently, the second layer was cast similarly over the previously cured layer after affixing the fiberglass sheet. The palm underwent a similar casting process, with injection molding for wedges and press molding for the skin, incorporating an intermediate step of attaching the fiberglass sheet. The wedges and skin were aligned and bonded by dispensing uncured Smooth-Sil 960 into the slits of the wedges and skin. The 3D-printed ASA interfaces were bonded to the cast fingers and palm by aligning them tightly and dispensing uncured Smooth-Sil 960 through slits in the interfaces. This ensured material flow through the cross beams, resulting in mechanical bonding once cured. Silicone tubes for vacuum actuation were attached to the respective parts by inserting and bonding them with uncured Smooth-Sil 960 at the inlets. While these fabrication steps were consistent across all competitions, additional unique features were incorporated based on the specific tasks outlined for each competition.

### 3.2 Competition specific fabrication

The initial gripper iterations used injection-molded suction cups fabricated using Smooth-Sil 940 in a two-part mold setup aided by compressed air (Figure 3C). The suction cup was centrally placed on the palm using a 3D-printed ASA holder. Ecoflex 30 acted as a soft padding between the fingertips and the nail tips. Using this configuration, the gripper could effortlessly lift large fruits such as watermelons and pick up thin, flat items like coins from the surface of a table. In RoboSoft 2022, the overall gripper design remained consistent with RoboSoft 2021, with the exception of replacing the soft active palm with a rigid 3D-printed ASA replica that had a fixed aperture.

For RoboSoft 2023, the gripper underwent various modifications, reducing its overall volume and eliminating the suction cup due to reduced payload capacity requirements. The number of wedges on each finger actuator decreased from five to three, and the palm size was proportionately reduced. Various nail types were considered, with the final choice being a curved hook-like nail with tapering thickness, proving effective for scooping noodles. Sensors were integrated at the fingers’ tips to maximize utility and functionality.

### 3.3 Sensor integration–RoboSoft 2023

**FIGURE 4:**
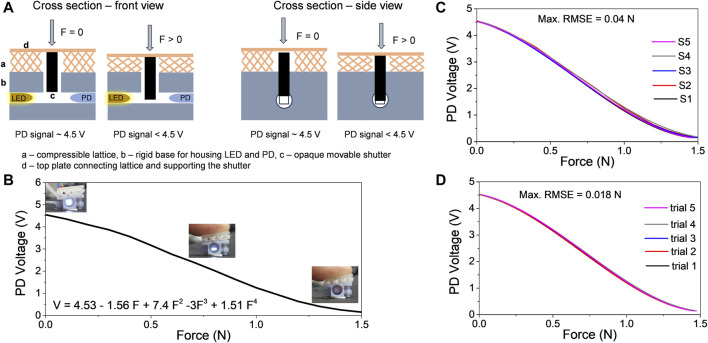
Attributes of the optical force sensor. **(A)** Working principle of the optical force sensor, **(B)** Plot of photodetector (PD) voltage of the sensor *versus* varying force when sensor is compressed (inset images show light intensity modulation by the shutter when force is applied), **(C)** plots of PD voltage *versus* force for five different sensor samples, **(D)** plots of PD voltage *versus* force of a sensor when tested 5 times.

For the optical force sensor integration, separate molds were meticulously designed for both the wedge structure and the skin of the finger (Figures 3D, E). The optical force sensor functioned as the uppermost wedge while being securely accommodated in the finger skin which was designed to align the sensor accurately. Simultaneously, the skin was cast in a manner that ensured the top surface of the optical force sensor seamlessly matched with the finger’s top surface. Special considerations were made for the skin, requiring cuts from the sides to accommodate an LED and a photodetector on either side of the sensor. Once the 3D-printed ASA molds were prepared, the wedges and skin underwent casting using a combination of injection molding and press molding, as detailed in earlier sections. The schematic of the design of the optical force sensor is shown in Figure 4A. The optical force sensor utilizes an opaque movable shutter anchored on a compressible lattice. The movable shutter modulates the intensity of the light passing from an LED to a photodetector (PD) depending on the compressed force. This principle is utilized to measure the force. The optical force sensor was 3D printed in Stratasys Objet Connex3 multi-material 3D printer. The 3D-printed samples were washed to remove the supports, followed by inserting the LED and the PD in the slots at the base. The sensor rigid base was equipped with cross beams for secure integration into the finger. To form a secure mechanical bond, the sensor was affixed to both the topmost wedge and the skin by dispensing uncured silicone into the sensor cross beams. The sensor was seamlessly integrated into the cavity of the finger through mechanical bonding. The placement of the sensor did not affect the functionality of the finger since it occupied the redundant space (a region that did not contribute to actuation) of the finger.

The Photodetector was connected to an LM393 sensor module that converted the photodetector signal into a voltage between 0 and 5 V depending on the light intensity. The sensor’s calibration curve was characterized using an IMADA ZTA-50 force gauge on an automated stage, operating at a rate of 4 mm/min. The gauge offered a resolution of 0.01 N. The sensor was compressed using a uniformly distributed load at a rate of 4 mm/min. During this compression, the force data was collected at an interval of 0.1 s while the photodetector voltage from the sensor was recorded via an Arduino Mega microcontroller at intervals of 0.1 s. The force *versus* photodetector voltage profile (Figure 4B) was plotted from the data collected from these experiments. When the above profile was fitted using a fourth-order polynomial, the following relationship was obtained:

$$V=4.53\;–\;1.56\;F\;–\;7.4\;F^{2}+3\;F^{3}+1.51\;F^{4}$$

To examine the consistency, five sensor samples were 3D-printed and their response to force was evaluated. Figure 4C depicts the PD voltage *versus* force plot of these five sensor samples. The maximum Root Mean Square Error (RMSE) among these samples was 0.04 N, indicating good repeatability across multiple samples. Additionally, five trials were conducted using one of the samples, and the maximum RMSE was found to be approximately 0.018 N, demonstrating reliable force measurement consistency over several trials (Figure 4D).

Similarly, for the tactile sensor, distinct molds were created for both the wedge and the skin of the finger (Figures 3D,E). In this instance, two prototypes were developed: one with the sensor entirely submerged in the wedge, ensuring no protrusion at the fingertip, and the second with a protrusion at the fingertip to accommodate the sensor plunger. Given the delicate nature of the tactile sensor, extreme care was exercised during handling and insertion of the sensor into the wedge. Once fitted into the wedge’s cavity, the sensor wires were cautiously threaded through the wedge’s holes. A drop of uncured silicone was carefully applied to the hole at the back of the wedge to secure the wires in place. Finally, the wedge with the sensor was inserted into the cast skin and bonded using uncured silicone. The design without a plunger protrusion was chosen for its greater robustness and reduced susceptibility to fluctuations caused by the slightest touch on the fingertip or movements. Tactile sensor fabrication and data collection process: The tactile sensor was fabricated by assembling several parts into a multi-layered architecture. A sensing tip that was cast in PDMS was bonded on the top of a stretchable substrate. Four strain sensors with ultra-high sensitivity were centrosymmetrically distributed around the sensing tip. In addition, this structure was sandwiched between two PET substrates and further put into a shell for the purpose of protecting the sensor and its circuit. Flexible printed circuits were used to connect the sensor to a data acquisition platform (Arduino). Extensive experimentation details along with a step-wise fabrication process have been provided in a previous work (Su et al., 2024).

**FIGURE 5:**
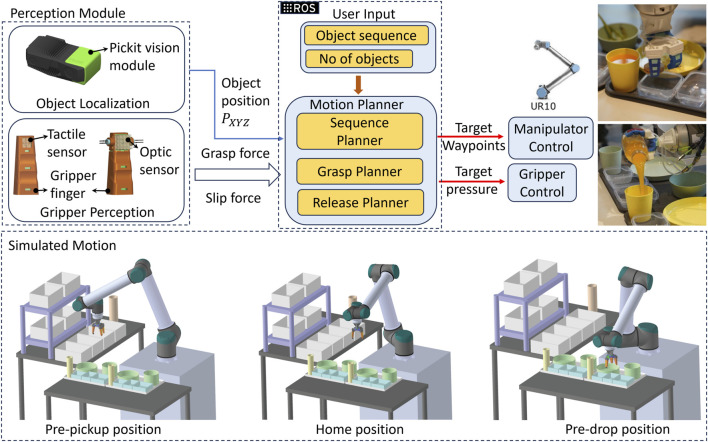
Schematic diagram showing various components integrated for autonomous grasping.

Two fingers were fabricated, one equipped with an optic sensor and the other with a tactile sensor (Figure 5). The finger with the optic sensor was positioned as a single finger opposite two others. This configuration was chosen because the overall grasping force on an object was influenced by the normal force acting on the fingers. In the case of dual fingers, this force was distributed, contingent on the grasping pose. Conversely, with a single finger, it represented a singular value. The optic sensor’s role was to regulate the grasping force for a specific object and to halt gripping when a predetermined threshold value was attained. In a controlled environment, specific vacuum levels for each item could be pre-programmed, eliminating the need for real-time force control. However, in this instance, as the item’s position was determined using a computer vision system, slight positional offsets were possible. The utilization of the optic sensor to manage the grasping force on the object thus became essential. Unlike a predetermined grasp that might miss the object due to aperture mismatch caused by a position offset, the optic sensor allowed gripping to persist until a secure hold was achieved starting from a wide aperture position that took care of any offsets. For all items in the list, predetermined force values were established—sufficient for a firm grasp to hold the object but restrained enough to prevent damage to delicate items. These values were then integrated into the overall program based on the order of grasping. In the case of the dual fingers, one finger was equipped with a tactile sensor. The tactile sensor’s function was to detect slip potential and prevent it by adjusting the robotic arm speed accordingly. Shear force values, indicating potential slips, were to be recorded for the items, and incorporated into the program, reducing the robot’s speed when these threshold values were detected during intermediate steps of robotic arm movement from the source table to the assembly table.

### 3.4 ROS integration–RoboSoft 2023

In our approach, the Pickit camera played a crucial role in object localization, providing coordinates that guided the entire path planning process implemented on ROS. The sequence of operations (Figure 5) unfolded seamlessly: first, the camera detected the top item (item 1) in the stack, relaying its coordinates to the ROS program. Subsequently, the robotic arm maneuvered to the pre-pick position of the container holding item 1. With precise coordinates from Pickit, the arm adeptly picked up the item, employing a gripping loop until the optic sensor registered a preset normal force threshold, ensuring a secure grasp. Upon reaching the threshold, a signal was dispatched to ROS to initiate the predetermined trajectory. The object was first moved to the pre-pick position and then moved to the assembly table, incorporating two intervals with a predetermined maximum arm speed. During these intervals, a check was conducted to ascertain whether the shear value of the tactile sensor fell within the predefined threshold. If within range, the arm maintained the same maximum speed; otherwise, it was halved as a precaution. Once positioned at the pre-drop location of the target container on the assembly table, the optical sensor’s voltage value was checked. A consistent reading indicated a secure grasp, while any alteration suggested a potential slip during trajectory between the tables. The item was subsequently securely deposited into its designated container at the assembly table, incrementing the respective item counter upon successful completion of this operation. The loop then seamlessly transitioned to the next item, repeating the entire process. This iterative and systematic approach ensured the accurate assembly of the required quantity for each item.

## 4 Experiments and results

### 4.1 RoboSoft 2021 entry

**FIGURE 6:**
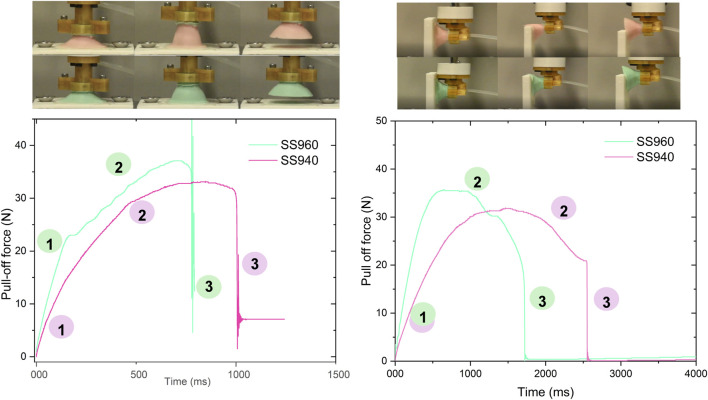
Pull off tests for suction cups of Smooth-Sil 940 and Smooth-Sil 960 on a horizonal base and a vertical base.

**FIGURE 7:**
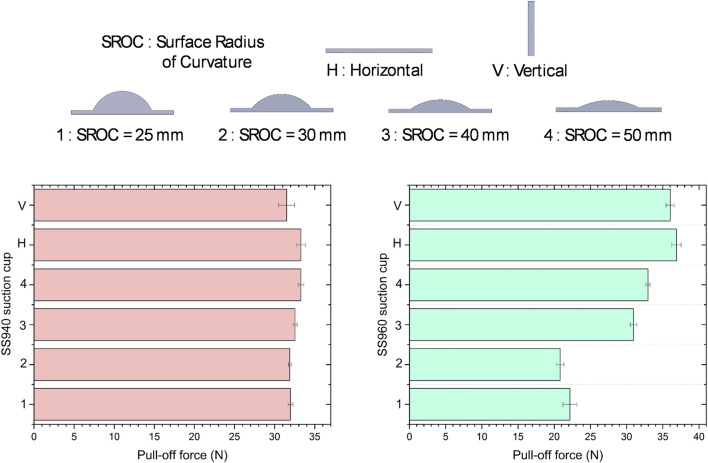
Pull off tests for suction cups of Smooth-Sil 940 and Smooth-Sil 960 on four surfaces with different radii of curvature.

Two material choices were considered for the fabrication of suction cup based on the purpose and payload capacity that is stiff Smooth-Sil 960 (Tensile Strength of 650 psi. and Maximum Elongation of 270%) and softer Smooth-Sil 940 (Tensile Strength of 600 psi. and Maximum Elongation of 300%). These choices were based on the hypotheses that stiff material helps to maintain the suction cup shape intact while vacuum is applied which could increase the payload capacity (due to comparatively more effective area being maintained under suction) however have the risk of shear failure (due to comparatively less conformity) in case of vibrations which were possible during pick and place robotic arm trajectory. Hence another choice of Smooth-Sil 940 which is comparatively softer, was considered and pull tests using IMADA ZTA-50 force gauge on an automated stage, were conducted to examine the suction forces of the respective suction cups. Two types of tests were conducted to determine the optimum choice. The first test involved pre-loading the suction cup up till a fixed deformation controlled by the automated stage, followed by measuring the pull-off force once a vacuum of −80 KPa was applied on a horizontal surface and a vertical surface. As shown in the Figure 6, the Smooth-Sil 940 suction cup performed comparably with the Smooth-Sil 960 in terms of pull-off force values, but was better in terms of the time of contact before breaking off which indicates a higher resistance to shear. This is because the softer cup provided more conformity in bending and accommodated the changes in suction cup shape without immediately breaking off the contact due to shear. The second test involved using surfaces of different radii of curvature and measuring the pull-off force in a similar sequence as before. As shown in the Figure 7, the Smooth-Sil 940 suction cup performed better compared to Smooth-Sil 960 suction cup for smaller radii of curvature surfaces (25 mm and 30 mm) indicating greater adaptability to the surface which is important to conform to fruit surfaces which could be irregular.

**FIGURE 8:**
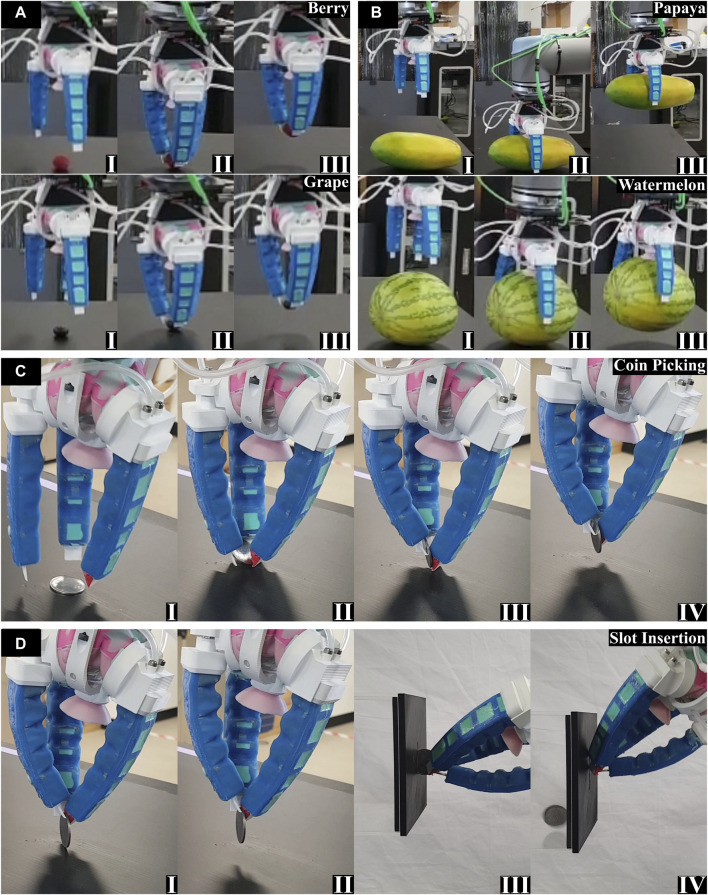
Completion of the tasks in RoboSoft 2021 competition (I, II, etc. denote the sequence of operations): **(A)** Task of picking a small delicate fruit exemplified by a raspberry and a single grape; **(B)** Task of lifting weightier items, such as watermelon and papaya; **(C)** Task of lifting a coin lying flat on the ground in a top-down approach; **(D)** Task of inserting the coin in the slot with additional manipulation steps.

The gripper designed for this scenario successfully executed all predetermined tasks within the day preceding the competition (Figure 8). Comprising fixed nails with soft Ecoflex 30 cushioning, an active soft palm, three soft fingers, and a central active suction cup, the fully assembled gripper weighed 250 g. Remarkably, it demonstrated the capacity to reliably lift items as heavy as 2 kg. As the competition adopted an online format due to COVID restrictions, the prescribed layout was replicated in our laboratory space, adhering to the guidelines outlined in the rulebook. The designated items were securely positioned and placed in a basket, set 50 cm apart—a distance the robotic arm (UR–10) was expected to traverse. The combination of fingernails and the precise aperture control of the soft palm effortlessly secured the first item, a small delicate fruit, exemplified by a raspberry and a single grape (Figure 8A). The collaborative grasping force generated by the suction cup, active palm, and fingers successfully lifted heavier items, such as a watermelon and a papaya (Figure 8B). Subsequently, the Ecoflex 30-assisted fingernails adeptly lifted a coin lying flat on a surface (Figure 8C). However, the coin’s orientation rendered it unsuitable for insertion into the coin slot, necessitating additional manipulation steps. The gripper and UR–10 were meticulously programmed to adjust the coin’s orientation, ensuring its alignment with the coin slot (Figure 8D). The coin insertion proved reliable during both the preliminary tests (refer Supplementary File Video S1) and the competition day, propelling our team to the semi-finals. Although the coin picking was successful in the semi-finals, a slight misalignment in coin positioning led to an insertion failure.

### 4.2 RoboSoft 2022 entry

**FIGURE 9:**
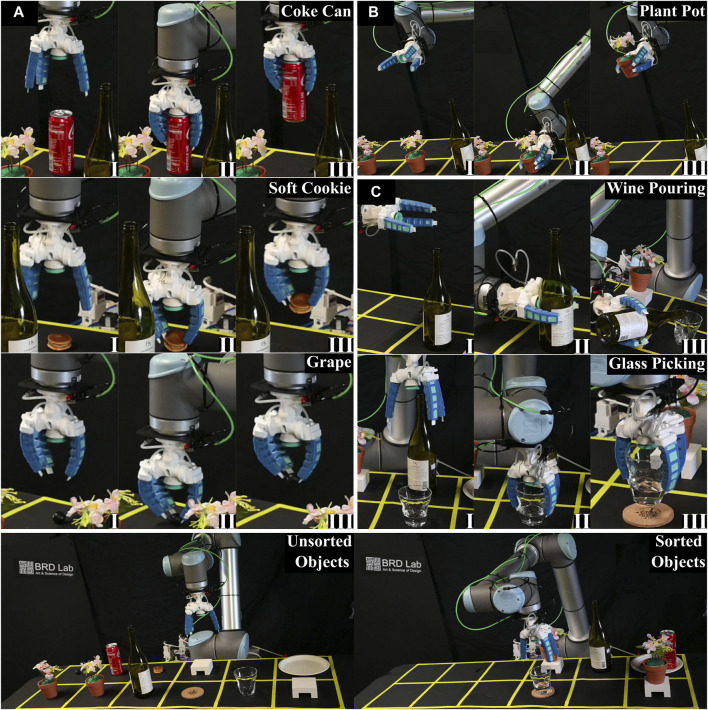
Completion of the tasks in RoboSoft 2022 competition (I, II, etc. denote the sequence of operations): **(A)** Task of handling a coke can, a soft cookie, and a single grape; **(B)** Task of handling plant pots; **(C)** Task of picking a wine bottle and pouring a dram of wine into the glass followed by positioning of the glass on the coaster.

The substitution of the active soft palm with a rigid, fixed-aperture palm 3D printed with ASA significantly mitigated vibrations, particularly when handling the wine bottle. A day before the competition, the gripper consistently grasped all items involved in the three tasks (Figure 9, Refer Supplementary File Video S1). During the handling of the 330 mL coke can, we observed that the side grasp lacked reliability, leading to occasional slips due to the can’s smooth surface. Recognizing this challenge, we leveraged the smooth top cover of the can, allowing the suction cup to be employed for secure grasping. This top-down approach, incorporating the suction cup on the palm and fingers on the side, reliably secured the coke can (Figure 9A). For the other two randomly selected items in the first task, a soft cookie and a single grape, the gripper adopted a partially actuated mode for aperture control, successfully grasping both items using its fingernails. In the second task, involving the handling of plant pots, the gripper approached the pots from the side and adeptly secured both without using the suction cup (Figure 9B). The fingers’ grip force alone proved sufficient for handling the pots effectively due to their tapered shape. In the final task of wine pouring, precise positioning and manipulation control were essential. The gripper approached the bottle from the side, activating the fingers once the bottle was within the aperture. The actuated fingers pushed the bottle against the suction cup, establishing a secure seal, after which the suction cup was activated. This sequence ensured a reliable grasp, enabling the manipulation of the bottle to pour a dram of wine into the glass before carefully placing the bottle back on the table (Figure 9C). Finally, the cup was positioned on the coaster by approaching the glass from the top. All waypoints and item positions were meticulously set on their predetermined locations, and the tasks were executed using the robotic arm UR–10’s waypoint programming. The entire set of tasks was completed in under 1 min and 20 s on the day of the competition, securing our team’s victory in the RoboSoft 2022 competition.

### 4.3 RoboSoft 2023 entry

The constructed gripper featured a soft active palm, a finger with a curved hook-shaped nail and an optical sensor for force control, and two opposing fingers with flat nails complemented by a tactile sensor embedded at one fingertip for slip detection. Prior to the competition, several images of the specified objects were captured, and corresponding libraries were created in the Pickit interface. Experimental trials were conducted to assess the reliability of object localization using a Pickit L-HD camera (refer Supplementary File Video S2). The camera accurately detected larger objects with a substantial form factor, such as sausages, meatballs, broccoli, and cookies. However, thin items like green beans and carrot slices remained undetectable, as the camera was designed for items with sizes of 50 × 50 × 10 mm and above.

**FIGURE 10:**
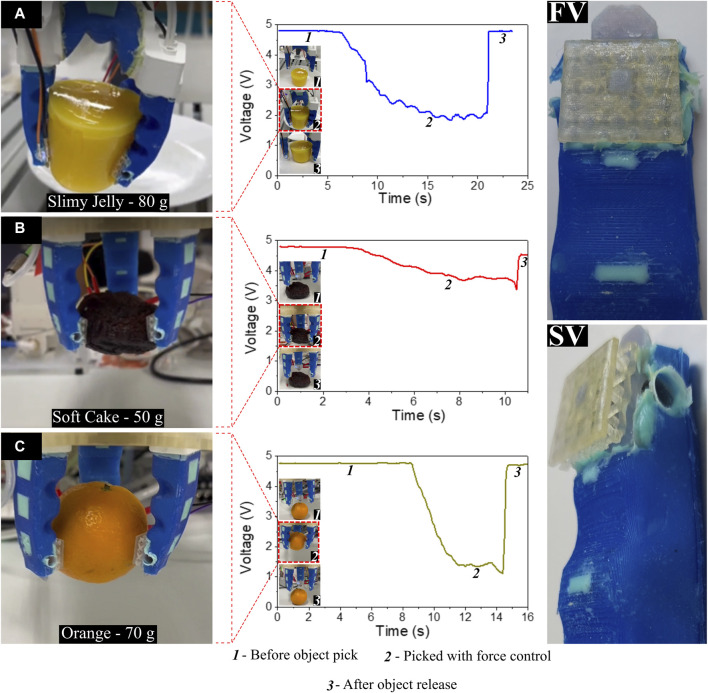
Experiments for obtaining the voltage values from light intensity of the optical sensor based on the grip force with front view (FV) and side view (SV) of the finger integrated with optic sensor. **(A-C)** Gripper handling a slimy jelly, a soft cake, and an orange with the corresponding graph showing the change in voltage values with time from picking up the item with force control while grasping and finally releasing the item.

An optical force sensor was employed to control the grasping force. The sensor operated based on the principle that the normal force on its surface would impact the light intensity transmitted from an LED to the photodetector on the opposite side. Converting the detected light intensity to a voltage signal allowed the derivation of a parametric relationship between force and voltage. The sensor shows an inverse linear relationship between the applied force (0–1.5 N) and the photodetector voltage (0–4.5 V) (Figure 4B). Further, the output of the sensor is a voltage signal between 0 and 5 V which can be directly attached to the analog input ports of the robot barring the need for complicated signal processing steps. These voltage values were crucial for determining the point at which the grasp became secure enough to lift an item without causing damage to soft objects. Specific experiments (Figure 10) were carried out for three items with varying stiffness—namely, the slimy jelly (refer Supplementary File Video S1), a soft cake, and an orange. These items represented the typical stiffness and weight encountered during the food tray assembly tasks of the competition. The test followed this protocol: (a) The gripper was positioned near the object (position one in the figures); (b) The gripper’s vacuum was activated, causing it to grasp the object, and the change in sensor signal was observed. The vacuum was increased at a rate of approximately 16 kPa per second, with the sensor signal recorded every 0.1 s and compared to a preset threshold. Once the sensor reached the preset threshold, the vacuum was maintained at a constant level (resulting in stable sensor signal at position two in the graph). The decrease in sensor signal from position one to position two in the graphs reflects this closed-loop feedback control of the vacuum based on the preset sensor signal. (c) After grasping the object, it was lifted and placed at the destination, and the vacuum was released. The abrupt decrease in sensor signal at position three in the graph corresponds to this action. Thus, the sensor played a crucial role in achieving predetermined grasp force through closed-loop control, detecting object grasp and release, and identifying slip (sudden drop in signal during pick and place) in case of unsuccessful grasps during the grasping action to be accounted in the final count. As observed from Figure 10, the threshold voltage (hence the force) required to safely grasp the object and perform pick and place operation depended on weight and stiffness of the object. Other key attributes such as surface roughness and the shape of the object also played a key role. The photodetector value required to achieve safe grasping for each of these objects were respectively: slimy jelly (80 g)–2 V; soft cake (50 g)–3.7 V; orange (70 g)–1.5 V.

The preset sensor signal for picking each object for the competition was determined through multiple trial-and-error experiments, where different sensor presets were tested to assess the quality of grasp (success, failure, excessive force) for each object. This data was then extrapolated to estimate thresholds for other objects that were encountered on the day of the competition considering their weight and roughness. For example, based on the sensor threshold data for the orange (relatively rigid), thresholds for cookies, juice cans, and carrots were estimated. The sensor threshold for the mango jelly was used to estimate thresholds for eggs and sausages, while data from the cake was used for broccoli. For these estimates an additional 10% force as a safety margin was chosen to ensure secure grasp (which could result in slight object damage, but in the competition, damaged pick-and-place attempts scored better than no pick).

**FIGURE 11:**
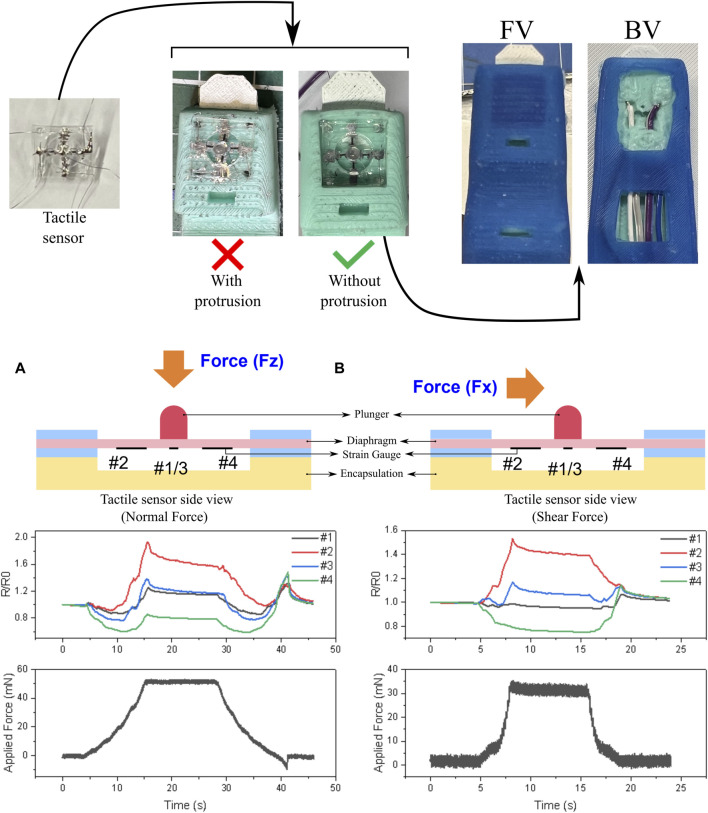
Fabrication of the finger integrated with the tactile sensor with the front view (FV) and the back view (BV) along with experiments to detect normal and shear forces on the fingertip: **(A)** Side view of the tactile sensor with labeled parts along with the graph showing the change in relative resistance of respective strain gauge with an application of normal force; **(B)** Side view of the tactile sensor with labeled parts along with the graph showing the change in relative resistance of respective strain gauge with an application of shear force.

Tactile sensors that can differentiate normal and shear force are of great importance to the dexterity of soft grippers. For example, a gripper is not able to perform grasping force optimization and slip prevention without a tactile sensor that can detect shear force. A tactile sensor comprising four sensitive strain gauges (sensor #1, #2, #3, and #4) was designed and fabricated that could differentiate normal and shear force. In our previous publication, an analytical and numerical modal to guide the design of tactile sensor was introduced (Su et al., 2024). This is a universal strategy to design and fabricate tactile sensors that can differentiate normal and shear forces and similar mechanisms to detect normal and shear forces have also been reported by (Harada et al., 2014; Won et al., 2019; Gao et al., 2023). This further proves the universality and repeatability of our approach. Following the completion of tactile sensor integration, experiments to detect normal and shear forces on the fingertip were conducted (Figure 11). There were four strain sensors (sensor #1, #2, #3 and #4) with ultra-high sensitivity that were centrosymmetrically distributed around the sensing tip. In addition, this structure was sandwiched between two PET substrates and further put into a shell for the purpose of protection of sensors and circuits. Furthermore, this sensor can be well integrated on a soft gripper due to its softness and compactness. It is worth noting the benefit from this geometrical distribution, that the spatial force applied on the sensing tip results in the deformation of the substrate. Such spatial deformation was reflected by the resistance change of the four strain sensors. For example, when the normal force was applied, the strain distribution around the sensing tip tends to be similar. Thus, resistance increases upon the normal force stimuli for all the four sensors. By contrast, the strain distribution was not even upon shear force stimulation, as indicated by the differences in the variation of resistance of the four strain sensors. Using this data, plans were made for further experiments to determine the respective maximum robotic arm speeds at which slip did not occur. However, these plans were not realized due to lack of time and are kept for future exploration. These speed values were to be established for the respective items specified in the list of RoboSoft 2023 competition.

The designed gripper featured a single curved nail due to the constraint that incorporating all inward curved nails would reduce the overall gripper’s aperture, rendering it insufficient for handling the disclosed cookie, a challenge revealed just 1 day before the competition. Nevertheless, the single curved nail proved advantageous, serving a dual purpose in scooping noodles and acting like a hook for other items. The overall integration, incorporating Pickit for localization and the optical sensor for force control was detailed in the motion planning script. However, the final positions could only be programmed on the preceding day of the competition.

**FIGURE 12:**
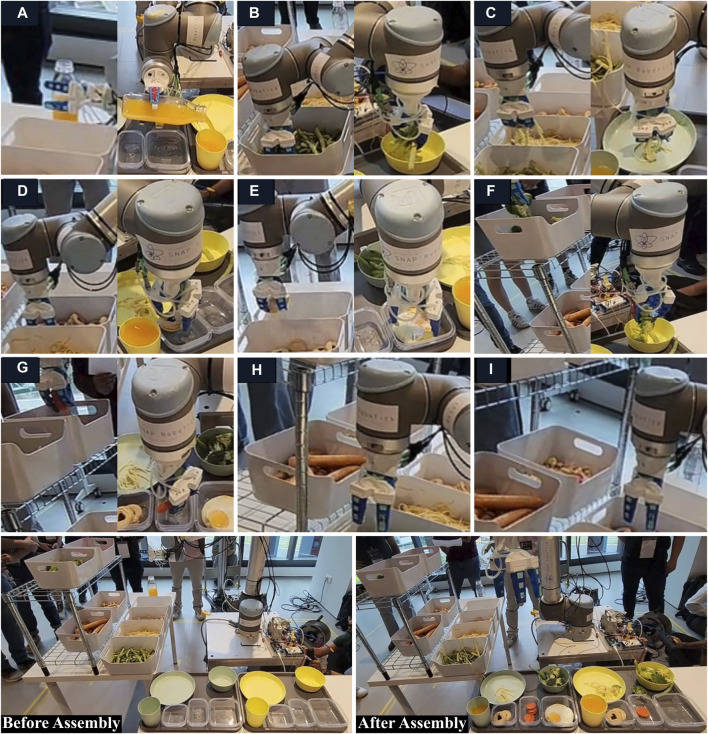
Completion of the tasks in RoboSoft 2023 competition (I, II, etc. denote the sequence of operations): **(A)** Task of handling the bottle of orange juice from the source table and pouring juice into the container at assembly table; **(B)** Task of picking green beans from the container at source table and placing in the container at assembly table; **(C)** Task of scooping noodles from the container at source table and placing on the plate at assembly table; **(D)** Task of picking cookies from the container at source table and placing in the container at assembly table; **(E)** Task of picking fried eggs from the container at source table and placing in the container at assembly table; **(F)** Task of picking broccolis from the container at source table and placing in the container at assembly table; **(G)** Task of picking sliced carrot pieces from the container at source table and placing in the container at assembly table; **(H, I)** The gripper along with wrist of the robotic arm could not approach the containers with sausages and ice gems due to limited space between the racks.

On the competition day, variations in lighting conditions caused a slight offset in item localization using the Pickit camera. Although this offset could be corrected using a pre-programmed offset difference in the final script, it proved inconsistent for different items. Additionally, due to their smaller form factor, some items could not be detected by the camera, and a highly recognizable item, the sausage, was inadvertently placed in a container on the bottom rack, obstructed from view by top containers (Figure 12H). Since all item final locations were disclosed only the day before the competition, the Pickit camera could only have been used with some offsets for the cookies, fried eggs, and broccoli. However, employing a depth camera presented certain drawbacks, notably increased time consumption as the camera first scanned for the topmost item in the stack before the robotic arm moved to that location. Consequently, we opted to abandon the use of the camera just before the competition and proceeded with waypoint programming. The trajectory was already confined to specific locations such as the bottle, and all container positions were fixed, with predetermined item pre-pick and pre-drop positions. To enhance grasp probability, each container at the source table was subdivided into sections, and for each pick, the arm was programmed to move to a designated section. The number of sections was tailored based on the quantity of items to be picked for each specific object in the list.

On the day of the competition, we experienced an intermittent issue with the input/output (I/O) usage of the UR10 controller which frequently caused abrupt disruption to the ROS program, often requiring the program to restart. We suspected that it might have to do with the fact that the manipulator that we used for the competition was an old UR10 that needs to use a deprecated ROS driver that has not been maintained and well tested anymore (GitHub, 2022). We tried to debug it but given the limited time and non-deterministic nature of the issue which could not be reproduced reliably, we were unable to pinpoint the issue promptly. Hence, a strategic decision not to use the optical sensor was made as these interruptions would have led to penalties in the scoring. Consequently, we opted to forgo the sensor for grasp force control and for counting to further reduce the time for complex processing, decision-making, and execution. This decision was informed by our observation that most items were sufficiently firm and not easily damaged by the gripper’s soft fingers. To account for any uncertainties such as slip and damage, we implemented a safety measure for each item. For instance, if the task involved grasping three cookies, we programmed the system to handle four cookies. On the day of the competition, our team successfully completed the entire set of tasks for two tray assemblies in under 16 min and 30 s, ultimately securing victory in the RoboSoft 2023 competition (Figure 12).

## 5 Discussion and lessons learnt

The three grippers employed in the IEEE RoboSoft competitions featured soft, vacuum-actuated hybrid fingers designed to seamlessly adapt to diverse objects (Table 3). Evolving from this foundation, the gripper design was tailored to the specific tasks, necessitating a customized multimodal approach with additional features and modes. Our team demonstrated adaptability by advancing to the semi-finals of RoboSoft 2021 and clinching the first prize in both 2022 and 2023 competitions, effectively navigating through evolving scenarios and overcoming challenges. The simulated scenarios mirrored unstructured dynamic environments, resembling industrial, domestic, or business setups where automated solutions with robotic hands interact with a wide array of items. In this context, soft robots play a pivotal role in expediting the transition to automation. These competitions not only served as a showcase for diverse soft robotic solutions but also facilitated benchmarking capabilities and embraced a peer-to-peer learning methodology.

**TABLE 3:** Summary of grasping tasks in all RoboSoft competitions Legend: Task Completed successfully ✓✓ Partially Completed ✓ Unsuccessful ×.

		Lab Trials	Finals	Object type	Actuation mode used
**Tasks (2021)**					
Watermelon pick and place		✓✓	✓✓	Large, heavy	Suction + Wide
Raspberry pick and place		✓✓	✓✓	Small, soft	Pinch
25 mm coin pick		✓✓	✓✓	Small, thin, flat	Pinch
25 mm coin placement in a coin slot		✓✓	×		
**Tasks (2022)**					
The pick and place	Coke can	✓✓	✓✓	Medium, cylinder	Suction + Power
	Marshmallow	✓✓	✓✓	Small, soft, delicate	Pinch
	Grape	✓✓	✓✓	Small, delicate	Pinch
Plant problem	Plant1	✓✓	✓✓	Medium, frustum	Power
	Plant2	✓✓	✓✓	Medium, frustum	Power
Whisky problem - Pouring and serving	Pouring	✓✓	✓✓	Medium, cylinder	Suction + Power
	Safe glass-placement	✓✓	✓✓	Medium, frustum	Power
**Autonomy**		×	×		
**Tasks (2023)**					
Sausages		×	×	Small, long, cylinder	Power
Broccoli		✓✓	✓✓	Small, delicate	Pinch
Carrots		✓✓	✓	Small, thin, flat	Pinch
Green Beans		✓✓	✓	Small, long, thin	Pinch
Spaghetti Noodles		✓✓	✓	Soft delicate strands	Scoop
Cookies		✓✓	✓	Small, thin, flat	Pinch
Fried eggs		✓✓	✓	Medium, soft, flat	Pinch
Ice gems		×	×	Small, soft, granular	Scoop
Orange juice		✓✓	✓✓	Medium, cylinder	Power
**Autonomy**		✓✓	×		

The winning team in RoboSoft 2021 utilized teleoperation for picking up and inserting the coin into the slot, allowing them to adapt to real-time changes. In contrast, our approach of waypoint programming demanded precision in placement. In RoboSoft 2022, autonomy became imperative, although not explicitly specified. On the day of the competition, random placement of objects was required. Despite receiving penalties for fixing object positions, our team secured victory by completing all tasks in record time. Other teams using automated setups faced significant delays and struggled to complete tasks within the allocated time. The suction cup complimented by the adaptable soft fingers played a crucial role, contributing to the successful lifting of heavy fruits in 2021 and facilitating wine pouring in 2022. Both competitions were conducted online due to COVID restrictions, with certain items disclosed a day prior. Replicating layouts in the laboratory enabled practice experiments, facilitating iterative improvements in gripper designs.

In RoboSoft 2023, our team endeavored to achieve full system automation. The competition unfolded in a single day, preceded by a day of testing on the arena, allowing teams to assess their robots on each task. Despite successful detection of some items in lab experiments, the vision system faced challenges in localization during the competition, prompting us to abandon the use of the camera. Further, we do wish to highlight that during the competition, some items (like broccoli) were picked in excess, while others (noodles and carrots) were picked in insufficient quantities, resulting in a reduced overall score. Improved performance could have been achieved if the vision system assisted in counting grasped objects, and sensor data was utilized to confirm successful grasps. However, the reliance on sensors for grasp control and motion planning was observed to add excessive execution time per each running loop, resulting in lower chances to pick up enough items within the required time frame. Instead, leveraging on the effectiveness of our versatile gripper and strategic approaches to enhance the grasp success rate, we achieved the highest score among all teams, securing the top prize in the competition. This competition underscored the significance of autonomy while highlighting its potential to slow down the system, emphasizing the need for a balanced approach to prevent over-engineering.

The base design of the vacuum-actuated multi-material hybrid gripper was modified to suit the requirements of three different competitions. The aspect of building on top of a technology is crucial in terms of developing a gripper that can do successful grasping of the different components in the respective competitions that can be extended beyond competitions to commercialization. The soft gripper as such is not a closed-loop solution and only after integrating with other components can be used in practical applications. The approach followed in our work to integrate the different components to suit the need is also impactful for taking a technology from lab to market. This was evident in the case of RoboSoft 2023 when the soft gripper had to be integrated with two types of sensors and other commercial products such as Pickit camera and a UR10 to offer it as a versatile autonomous grasping solution for food assembly. All three soft grippers performed reliably, executing numerous tasks both in the competition and under laboratory conditions. This success instilled confidence in their reliability and robustness. Looking ahead, we anticipate more formidable challenges, such as handling gravy or semi-solid items, for which our group has developed another iteration named the reconfigurable workspace soft gripper (Jain et al., 2023). Ongoing work involves the integration of features like hydrophobic surfaces on fingertips and soft, flexible interconnects, enhancing sensor integration capabilities (Jain et al., 2020b; Plamootil Mathai et al., 2022; Hegde et al., 2023). Finally, the development of a hybrid multimodal gripper, incorporating both rigid and soft components, necessitates a distinct fabrication approach. This can be extended by collaborating with even commercial partners to come up with viable fabrication techniques that could act as a bridge in integrating different components. To overcome these challenges, future studies should also delve into advanced fabrication techniques, leveraging multi-material printing for a more streamlined process (Joseph et al., 2021; Calais et al., 2022). Moreover, efforts should also focus on seamlessly integrating vision and sensor data to deploy a reliable solution that is both robust and efficient.

## Data Availability

The original contributions presented in the study are included in the article/Supplementary Material, further inquiries can be directed to the corresponding author.

## Footnotes

1 IEEE ICRA 2023 Competitions https://www.icra2023.org/competitions

2 IEEE RoboSoft Competition https://softroboticsconference.org/robot-competition-proposals/

## References

[B1] Alici G. Richards L. (2023). “A locking mechanism for a multipurpose gripping system for unmanned aerial vehicles,” in Proceedings of the 2023 6th International Conference on Advances in Robotics, New York, NY, USA, July 5 - 8, 2023 (Association for Computing Machinery).

[B2] Amend J. Lipson H. (2017). The JamHand: dexterous manipulation with minimal actuation. Soft Robot. 4 (1), 70–80. 10.1089/soro.2016.0037 29182098

[B3] Artemiadis P. Thalman C. (2020). A review of soft wearable robots that provide active assistance: trends, common actuation methods, fabrication, and applications. Wearable Technol. 1, e3. 10.1017/wtc.2020.4 PMC11265391 39050264

[B4] Calais T. Sanandiya N. D. Jain S. Kanhere E. V. Kumar S. Yeow R. C. H. (2022). Freeform liquid 3D printing of soft functional components for soft robotics. ACS Appl. Mater Interfaces 14 (1), 2301–2315. 10.1021/acsami.1c20209 34962370

[B5] Caldwell D. G. Davis S. Moreno Masey R. J. Gray J. O. (2009). “Automation in food processing,” in Springer handbook of automation. Editor Nof S. Y. (Berlin, Heidelberg: Springer Berlin Heidelberg), 1041–1059. 10.1007/978-3-540-78831-7_60

[B6] Chin L. Barscevicius F. Lipton J. Rus D. (2020). “Multiplexed manipulation: versatile multimodal grasping via a hybrid soft gripper,” in 2020 IEEE International Conference on Robotics and Automation (ICRA), China, 31 Aug, 2020 (ICRA), 8949–8955.

[B7] Choi M.-S. Lee D.-H. Park H. Kim Y.-J. Jang G.-R. Shin Y.-D. (2017). “Development of multi-purpose universal gripper,” in 2017 56th Annual Conference of the Society of Instrument and Control Engineers of Japan (SICE), USA, 19-22 Sept. 2017 (SICE), 1421–1424.

[B8] Downs A. Kootbally Z. Harrison W. Pilliptchak P. Antonishek B. Aksu M. (2021). Assessing industrial robot agility through international competitions. Robotics computer-integrated Manuf. 70, 102113. 10.1016/j.rcim.2020.102113 PMC10092066 37056680

[B9] Elfferich J. F. Dodou D. Santina C. D. (2022). Soft robotic grippers for crop handling or harvesting: a review. IEEE Access 10, 75428–75443. 10.1109/access.2022.3190863

[B10] Fitzgerald S. G. Delaney G. W. Howard D. Maire F. (2021). “Evolving soft robotic jamming grippers,” in Proceedings of the genetic and evolutionary computation conference (New York, NY, USA: Association for Computing Machinery), 102–110. (GECCO ’21). Available from:. 10.1145/3449639.3459331

[B11] Gao Y. Huang X. Mann I. S. Su H. J. (2020). A novel variable stiffness compliant robotic gripper based on layer jamming. J. Mech. Robotics 12 (051013). 10.1115/1.4047156

[B12] Gao Y. Zhang B. Liu Y. Yao K. Huang X. Li J. (2023). Mechanoreceptor inspired electronic skin for multi-modal tactile information decoding. Adv. Mater. Technol. 8 (1), 2200759. 10.1002/admt.202200759

[B13] Gariya N. Kumar P. (2022). A comparison of plane, slow pneu-net, and fast pneu-net designs of soft pneumatic actuators based on bending behavior. Mater. Today Proc. 65, 3799–3805. 10.1016/j.matpr.2022.06.576

[B14] GitHub (2022). GitHub. Available at: https://github.com/ros-industrial/ur_modern_driver/commits/master/.

[B15] Harada S. Kanao K. Yamamoto Y. Arie T. Akita S. Takei K. (2014). Fully printed flexible fingerprint-like three-axis tactile and slip force and temperature sensors for artificial skin. ACS Nano 8 (12), 12851–12857. 10.1021/nn506293y 25437513

[B16] Hegde C. Su J. Tan J. M. R. He K. Chen X. Magdassi S. (2023). Sensing in soft robotics. ACS Nano. 17 (16), 15277–15307. 10.1021/acsnano.3c04089 37530475 PMC10448757

[B17] Jain S. Dontu S. Teoh J. E. M. Alvarado P. V. Y. (2023). A multimodal, reconfigurable workspace soft gripper for advanced grasping tasks. Soft Robot. 10 (3), 527–544. 10.1089/soro.2021.0225 36346280 PMC10278002

[B18] Jain S. Stalin T. Kanhere E. Alvarado P. V. y (2020b). Flexible fiber interconnects for soft mechatronics. IEEE Robotics Automation Lett. 5 (3), 3907–3914. 10.1109/lra.2020.2982367

[B19] Jain S. Stalin T. Subramaniam V. Agarwal J. y Alvarado P. V. (2020a). “A soft gripper with retractable nails for advanced grasping and manipulation,” in 2020 IEEE International Conference on Robotics and Automation (ICRA), USA, 31 Aug, 2020 (IEEE), 6928–6934.

[B20] Jiao Z. Ji C. Zou J. Yang H. Pan M. (2019). Vacuum-powered soft pneumatic twisting actuators to empower new capabilities for soft robots. Adv. Mater. Technol. 4 (1), 1800429. 10.1002/admt.201800429

[B21] Joseph V. S. Calais T. Stalin T. Jain S. Thanigaivel N. K. Sanandiya N. D. (2021). Silicone/epoxy hybrid resins with tunable mechanical and interfacial properties for additive manufacture of soft robots. Appl. Mater. Today 22, 100979. 10.1016/j.apmt.2021.100979

[B22] Jungmittag A. Pesole A. (2019) The impact of robots on labour productivity: a panel data approach covering 9 industries and 12 countries. Seville: European Commission, Joint Research Centre JRC. Available at: http://hdl.handle.net/10419/231332.

[B23] Miron G. Bédard B. Plante J. S. (2018). Sleeved bending actuators for soft grippers: a durable solution for high force-to-weight applications. Actuators 7 (3), 40. 10.3390/act7030040

[B24] Plamootil Mathai A. R. Stalin T. Valvivia y Alvarado P. (2022). Flexible fiber inductive coils for soft robots and wearable devices. IEEE Robotics Automation Lett. 7 (2), 5711–5718. 10.1109/lra.2022.3159864

[B25] Pustavrh J. Hočevar M. Podržaj P. Trajkovski A. Majdič F. (2023). Comparison of hydraulic, pneumatic and electric linear actuation systems. Sci. Rep. 13 (1), 20938. 10.1038/s41598-023-47602-x 38016978 PMC10684514

[B26] Schmalz (2022). Design of the suction cup. Available at: https://www.schmalz.com/en/vacuum-knowledge/the-vacuum-system-and-its-components/vacuum-suction-cups/design-of-the-suction-cup/.

[B27] Shintake J. Cacucciolo V. Floreano D. Shea H. (2018). Soft robotic grippers. Adv. Mater. 30 (29), 1707035. 10.1002/adma.201707035 29736928

[B28] Su J. Zhang H. Li H. He K. Tu J. Zhang F. (2024). Skin-inspired multi-modal mechanoreceptors for dynamic haptic exploration. Adv. Mater., 2311549. 10.1002/adma.202311549 38363810

[B29] Subramaniam V. Jain S. Agarwal J. Valdivia y Alvarado P. (2020). Design and characterization of a hybrid soft gripper with active palm pose control. Int. J. Robotics Res. 39 (14), 1668–1685. 10.1177/0278364920918918

[B30] Tawk C. Gillett A. het P. M. Spinks G. M. Alici G. (2019). A 3D-printed omni-purpose soft gripper. IEEE Trans. Robotics 35 (5), 1268–1275. 10.1109/tro.2019.2924386

[B31] Wang Y. Yang Z. Zhou H. Zhao C. Barimah B. Li B. (2022b). Inflatable particle-jammed robotic gripper based on integration of positive pressure and partial filling. Soft Robot. 9 (2), 309–323. 10.1089/soro.2020.0139 34107751

[B32] Wang Z. Hirai S. Kawamura S. (2022a). Challenges and opportunities in robotic food handling: a review. Front. Robotics AI 8, 789107. 10.3389/frobt.2021.789107 PMC8794010 35096983

[B33] Wang Z. Kanegae R. Hirai S. (2021). Circular shell gripper for handling food products. Soft Robot. 8 (5), 542–554. 10.1089/soro.2019.0140 32822254

[B34] Won S. M. Wang H. Kim B. H. Lee K. Jang H. Kwon K. (2019). Multimodal sensing with a three-dimensional piezoresistive structure. ACS Nano 13 (10), 10972–10979. 10.1021/acsnano.9b02030 31124670

[B35] Zaidi S. Maselli M. Laschi C. Cianchetti M. (2021). Actuation technologies for soft robot grippers and manipulators: a review. Curr. Robot. Rep. 2 (3), 355–369. 10.1007/s43154-021-00054-5

[B36] Zhu J. Chai Z. Yong H. Xu Y. Guo C. Ding H. (2023). Bioinspired multimodal multipose hybrid fingers for wide-range force, compliant, and stable grasping. Soft Robot. 10 (1), 30–39. 10.1089/soro.2021.0126 35584255

